# Diphenyl-Methane Based Thyromimetic Inhibitors for Transthyretin Amyloidosis

**DOI:** 10.3390/ijms22073488

**Published:** 2021-03-28

**Authors:** Bokyung Kim, Young Ho Ko, Massimiliano Runfola, Simona Rapposelli, Gabriella Ortore, Grazia Chiellini, Jin Hae Kim

**Affiliations:** 1 Department of New Biology, Daegu Gyeongbuk Institute of Science & Technology (DGIST), Daegu 42988, Korea; bkkim@dgist.ac.kr; 2 Center for Self-Assembly and Complexity, Institute for Basic Science, Pohang 37673, Korea; yhko@ibs.re.kr; 3 Department of Pharmacy, University of Pisa, 56100 Pisa, Italy; massimiliano.runfola@farm.unipi.it (M.R.); simona.rapposelli@unipi.it (S.R.); 4 Department of Pathology, University of Pisa, 56100 Pisa, Italy

**Keywords:** transthyretin, thyromimetics, sobetirome, TTR amyloidosis, TTR stabilizers, protein aggregation

## Abstract

Thyromimetics, whose physicochemical characteristics are analog to thyroid hormones (THs) and their derivatives, are promising candidates as novel therapeutics for neurodegenerative and metabolic pathologies. In particular, sobetirome (GC-1), one of the initial halogen-free thyromimetics, and newly synthesized IS25 and TG68, with optimized ADME-Tox profile, have recently attracted attention owing to their superior therapeutic benefits, selectivity, and enhanced permeability. Here, we further explored the functional capabilities of these thyromimetics to inhibit transthyretin (TTR) amyloidosis. TTR is a homotetrameric transporter protein for THs, yet it is also responsible for severe amyloid fibril formation, which is facilitated by tetramer dissociation into non-native monomers. By combining nuclear magnetic resonance (NMR) spectroscopy, computational simulation, and biochemical assays, we found that GC-1 and newly designed diphenyl-methane-based thyromimetics, namely IS25 and TG68, are TTR stabilizers and efficient suppressors of TTR aggregation. Based on these observations, we propose the novel potential of thyromimetics as a multi-functional therapeutic molecule for TTR-related pathologies, including neurodegenerative diseases.

## 1. Introduction

Transthyretin (TTR) is an essential transporter of the thyroid hormone (TH) and a holo-retinol-binding protein. Under physiological conditions, TTR forms a ~55 kDa homotetrameric complex, on which two hydrophobic pockets are constructed as binding sites for thyroxine (T_4_) [1,2]. Notably, the quaternary stability of this protein is important not only for its physiological function but also to suppress its amyloidogenic propensity [3]. In its native tetrameric state, TTR is a non-amyloidogenic protein, whereas its dissociation into non-native monomers facilitates aggregation and formation of amyloid fibrils [4]. Due to the relative predominance of TTR in human serum and cerebrospinal fluid, TTR amyloidosis results in detrimental amyloidogenic diseases. For example, senile systemic amyloidosis is caused by the spontaneous aggregation of wild-type (WT) TTR, while familial amyloid polyneuropathy or familial amyloid cardiomyopathy is attributed to genetic modifications of TTR [5]. Indeed, more than 100 mutations have been reported to date, and it was shown that most pathogenic mutations facilitate fibril formation by reducing the stability of the TTR tetramer [6]. Recent investigations in the USA estimated that over three percent of the African American population had a specific TTR mutation that significantly increases the risk of TTR amyloidosis [7], while approximately eight percent of patients with suspected cardiac amyloidosis having pathogenic TTR mutations [8].

Therefore, researchers have developed several strategies for therapeutic intervention of TTR amyloidosis; e.g., liver transplantation procedures for hereditary TTR amyloidosis [9], and RNAi-based approaches to modulate the overall expression of TTR plasma protein [10]. Additionally, it was found that T_4_ binding to the hydrophobic pockets of TTR tetramer inhibited the acid-induced TTR aggregation; a set of thyromimetic molecules were designed as ‘kinetic stabilizers’ to repress monomer dissociation and fibril formation [11,12]. These efforts resulted in the development of tafamidis, a clinically proven drug for TTR amyloidosis [13]. However, it should be noted that the efficacy of tafamidis is limited due to its low permeability across the blood–brain barrier (BBB), and blood–ocular barrier; about 10% of TTR is produced at the choroid plexus and retina [14,15]. To develop novel therapeutics for TTR amyloidosis, therefore, several active investigations are currently ongoing. For example, tolcapone, the approved drug molecule for Parkinson’s disease, was shown to bind to TTR tetramer better than tafamidis and exhibit superior BBB permeability, thus being a strong therapeutic candidate for TTR amyloidosis [16,17]. In addition, the recent development of mds84, the bivalent binder occupying both T_4_ binding pockets simultaneously, has attracted attention because it has shown highly potent effects not only for tetramer stabilization but also for the prevention of TTR proteolysis [18]; the proteolysis-induced fragmentation of TTR was recently proposed as an important mechanism facilitating TTR amyloidosis [19,20].

Interestingly, along with its amyloidogenic property, TTR has also been an important target of active research for its neuroprotective function against amyloid-β (Aβ) aggregation. A series of studies have shown that TTR is a major binder of Aβ in cerebrospinal fluid and plays an essential role in suppressing Aβ aggregation in neurodegenerative pathologies [21,22,23]. Furthermore, it is suggested that TTR stabilizers can enhance the capability of TTR to suppress the cytotoxicity of Aβ and may function as a potential modulator of Aβ aggregation [24,25,26]. Recently, it was also reported that TTR interferes with the processing mechanism of Aβ precursor proteins [27], while the tetramer stabilization of TTR can be an effective strategy to reduce Aβ-related pathology [28]. Taken together, these observations implicate the intriguing potential of TTR and TTR stabilizers as novel targets for therapeutic intervention in Alzheimer’s disease.

**Figure 1:**
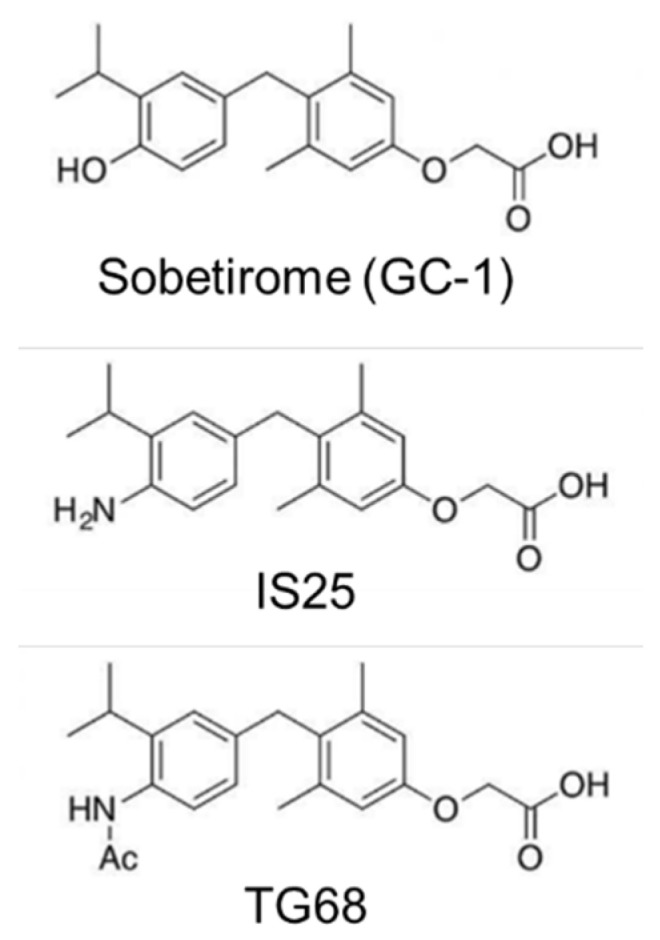
The thyromimetic molecules: sobetirome (GC-1), and its anologs, IS25, and TG68 [32].

On the other hand, although tafamidis does not seem to exhibit any other activity analog to hormonal molecules, thyromimetics have attracted attention for their therapeutic TH-like effects [29,30,31,32]. Diverse thyromimetics have been developed to modulate their activity, permeability, and selectivity, without hampering their therapeutic benefits [31,33]. In particular, a series of thyromimetics specific for TH receptor β (TRβ) demonstrated various advantageous features against neurodegenerative diseases and metabolic disorders, without the severe side effects often mediated by the activation of TH receptor α [32]. One of the initial thyromimetics designed for this purpose is sobetirome (GC-1) [34], which exhibited most of the beneficial effects of T_3_ against obesity and lipid metabolism disorder [35]. With peripheral injection, GC-1 was shown to accumulate in the liver, and also in significant amounts in the brain, proving its enhanced permeability across the BBB [36,37]. Notably, this molecule along with its subsequent analogs, such as eprotirome, underwent clinical trials, though it was discontinued despite promising results without any obvious deleterious effect. Since its initial development, many analogs of GC-1 have been designed and tested for functionality, selectivity, and permeability [38,39]. In particular, IS25 and TG68 (Figure 1) showed encouraging off-target and ADME-Tox profiles both in vitro and in vivo [40,41], facilitating liver proliferation and alleviating lipid metabolism disorders, without exhibiting T_3_-induced cardiotoxicity [42].

In the present study, we tested whether GC-1, IS25, and TG68, have additional functionality as binders of TTR tetramer, inhibiting its aggregation. Indeed, the interaction of GC-1 with TTR and its suppressive activity against TTR amyloidosis was reported once [43], yet it was not followed by further studies to elucidate its detailed molecular mechanism and to expand chemical diversity and functionality of GC-1. To this end, we first investigated whether the selected thyromimetics were able to suppress TTR amyloidosis. Subsequently, we characterized the interaction of TTR with these molecules, using nuclear magnetic resonance (NMR) spectroscopy, computational docking simulation, and molecular dynamics calculations. Our observations provide strong evidence that the thyromimetic ligands tested here are highly attractive candidates as potent inhibitors of TTR amyloidosis.

## 2. Results

**Figure 2:**
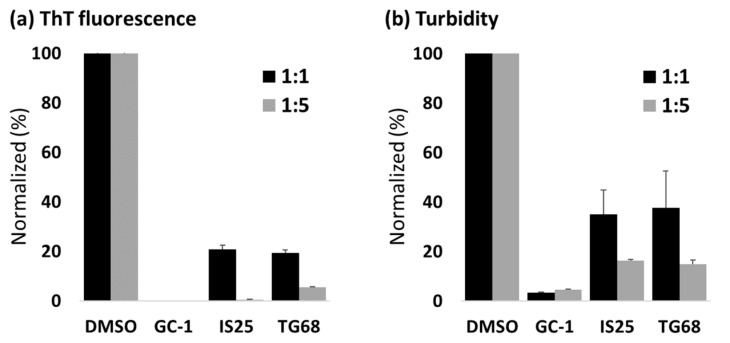
Thioflavin T (ThT) fluorescence (**a**) and turbidity (**b**) assay results of WT TTR in the presence of GC-1, IS25, or TG68, or absence (DMSO). The samples for both assays were incubated in pH 4.2 at 37 °C for four days. The data is normalized to the DMSO control. Results are given as mean ± SD (*n* = 3).

To validate the efficacy of thyromimetic molecules for TTR amyloidosis, we first tested whether GC-1, IS25, and TG68, are able to repress the aggregation propensity of TTR. We conducted an acid-induced aggregation assay with WT TTR in the absence or presence of the test compounds [4,44]. The concentration of the test compounds in the aggregation mixture were either equimolar or five-fold; the previous study reported that the level of GC-1 in a rat model could reach as high as 53 μM [37], suggesting that TTR may encounter the excess amount of GC-1 even in a physiological condition. Amyloid formation in the incubated samples was quantified with thioflavin T (ThT) fluorescence and turbidity measurements [45]. The results showed that all three compounds were able to inhibit TTR aggregation efficiently (Figure 2). In particular, the inhibition effect of GC-1 displayed complete, and near-complete suppression of ThT fluorescence and turbidity, respectively.

**Figure 3:**
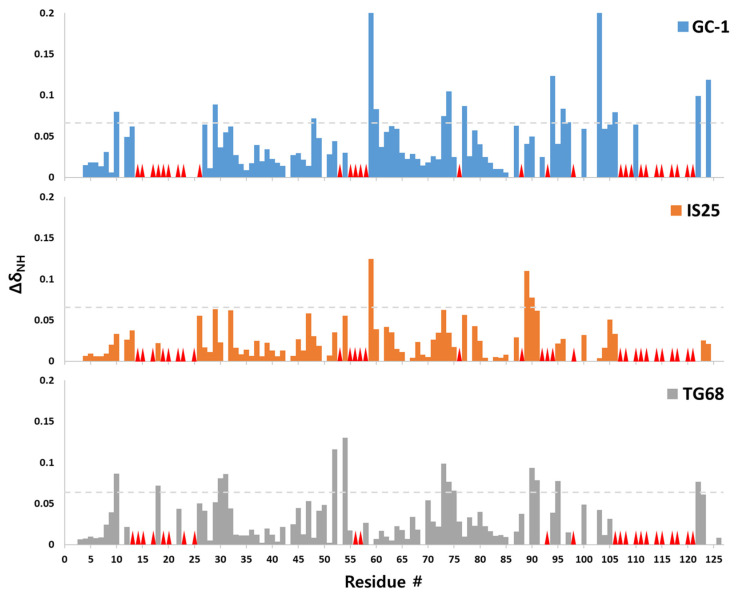
^1^H-^15^N backbone signal perturbation plot of WT TTR upon addition of GC-1, IS25, and TG68. The signal perturbations are calculated from the following equation: Δδ_NH_ = [(Δδ_N_)^2^/5 + (Δδ_H_)^2^]^1/2^, where Δδ_N_ and Δδ_H_ are the chemical shift differences of the ^1^H-^15^N signals between the free and ligand-bound states of WT TTR. The red triangles indicate the residue whose ^1^H-^15^N backbone signal disappeared by the addition of ligand molecules. The residues whose signals could not be assigned before or after ligand addition were left blank in the plot. The gray dotted line was placed at the value of 0.065, which was the overall mean plus SD.

The aggregation assay results indicated that the compounds bound to TTR and stabilized its tetrameric state. To further investigate their interactions, we employed NMR spectroscopy. NMR spectroscopy is a powerful technique that provides residue-specific information for diverse biomolecular interactions. As the tetrameric complex of WT TTR has a molecular size of ~55 kDa, partial deuteration, along with ^13^C and ^15^N-labeling procedures, was performed to obtain the sufficient resolution and sensitivity of the signals. Subsequently, GC-1, IS25, and TG68, dissolved in deuterated dimethyl sulfoxide (DMSO-d_6_) were titrated to the WT TTR sample, and 2D ^1^H-^15^N HSQC NMR spectra were obtained to monitor perturbations of WT TTR signals (Figure S1). For certain mixture samples, a 3D HNCA spectrum was acquired to resolve the overlapped signals in the 2D spectrum. Thereafter, the perturbation of backbone ^1^H-^15^N signals was plotted to evaluate the effects of ligand binding to WT TTR (Figure 3). Notably, a few ^1^H-^15^N signals broadened beyond detection upon ligand addition. We considered the corresponding residues to be the most affected by interaction with ligand molecules. In addition, certain ^1^H-^15^N signals showed significant shifts, indicating that the corresponding residues were highly affected by the interaction with the ligand molecules. Finally, a considerable number of ^1^H-^15^N signals did not show significant perturbation during the titration, and these residues were deemed not to be directly involved in the interaction. Overall, it was evident that GC-1 induced the most significant perturbation to the WT TTR signals, with the effects of IS25 and TG68 being less noticeable. This observation is consistent with the aggregation assays, where the inhibitory effect of GC-1 on TTR amyloid formation was also the most efficient among the thyromimetics tested. 

The signal perturbation pattern was mapped onto the structural model of WT TTR (PDB 4TLT [46]; Figure S2). From the previous X-ray crystallographic studies of T_4_-, GC-1-, and tafamidis-bound TTR (PDB 2ROX [2], 3NEE [43], and 3TCT [13], respectively), it was commonly observed that the TTR residues M13, K15-L17, E54, T106, A108-L110, S117-T119, and V121 were directly involved in the ligand interaction. The NMR signal perturbation results were consistent with the X-ray crystallographic observations; the signals corresponding to the residues mentioned above either significantly broadened or shifted due to the addition of GC-1, IS25, or TG68. However, there were also notable discrepancies between the two observations. Our results showed that the residues that are somewhat far from the ligand-binding site, such as A19-A25, G53, L55-T59, K76, H88, E92-V94, and N98, were significantly perturbed by the ligand interaction. Interestingly, although previous NMR spectroscopic studies investigating the interaction between TTR and tafamidis reported consistent signal perturbation [18,47], it seems that tafamidis did not induce noticeable signal perturbation for the residues G53-T59, indicating that GC-1 and the related compounds constitute a novel interaction network in this region of TTR (see below for details).

Subsequently, a computational docking simulation was employed to further explore the interaction between our thyromimetic ligands and TTR. The X-ray crystallographic structural models of the T_4_– and GC-1–TTR complexes (PDB 2ROX [2] and 3NEE [43], respectively) were examined in view of validating and preparing structural models for docking simulations. Although the two complexes present an overall RMSD of 0.3 Å, their hydration patterns differ significantly. The structural model of the GC-1–TTR complex (PDB 3NEE) showed the inclusion of a water molecule (numbered 162 in the chain A) in the binding site, which formed hydrogen bonds with several key residues for ligand binding, such as A108, S117, and T119. The similar water molecule was frequently found in the apo form (e.g., PDB 4TLT [46]), and sometimes observed as in the case of the flavonoid ligand-bound state [48], while it was often displaced by the ligand binding (e.g., PDB 3TCT [13]). The structural model of the T_4_–TTR complex (PDB 2ROX) does not have a water molecule occupying the same site with the GC-1–TTR complex. Upon the docking trials with the structural models of GC-1–TTR and T_4_–TTR complexes, we found that the best docking poses of GC-1 converged to a highly similar conformation, regardless of their original states, with a displacement of approximately 2 Å RMSD from the 3NEE structural model (see Figure S3a). Additionally, the docking poses of IS25 and TG68 (Figure S3b,c) that were obtained from the 2ROX and 3NEE structures were very similar. However, as a few simulation trials with the 3NEE structure suffered from unreliable results of some atoms modeled with zero occupancy in the protein core, we decided to continue our study with the 2ROX structure. We also speculated that 2ROX is more appropriate as a starting model for the consistent calculation of the ligand-bound TTR complexes. The results of the calculations indicated that all compounds were capable of adopting a good interaction mode (Figure S4). The complexes showed that one end (the carboxylate) was able to form an ionic interaction with the terminal amine of K15, and a hydrogen bond with T106, while the opposite end (the hydroxyl, amine, and amide groups of GC-1, IS25, and TG68, respectively), formed a polar interaction with residue S117, which was very effective for GC-1 (mediated by a water molecule in the crystal structure), and IS25, though weaker for TG68. The hydroxyl group of GC1 was involved in a hydrogen bond with the backbone of this residue, whereas the aniline of IS25, and the carbonyl of TG68 were less inclined to form a hydrogen bond, thereby exposing their distal polar group to the S117 side chain. S117 is located deep inside the hydrophobic cavity, with which efficient TTR stabilizers such as tafamidis and iododiflunisal interact [13,49]. E54 also indirectly participated in this interaction by mediating an ionic interaction with K15, thus contributing to the further stabilization of the complex.

**Figure 4:**
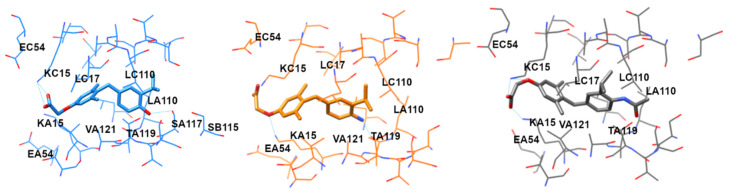
Structure of GC-1 (blue), IS25 (orange), and TG68 (gray), in the complex with TTR after MD simulation. The hydrogen bonds were denoted as cyan lines, and the residues from different chains (monomeric subunits) of the TTR tetramer were denoted with the middle letter, A through D.

A further molecular dynamics (MD) simulation was carried out to test the stability of our complexes and overcome the protein rigidity in the docking calculation, where only S117 residues were free to assume alternate torsion position, detected in many TTR crystal structures. MD simulations induced slight modifications in the GC-1 complex, whereas in TG68–, and especially in the IS25–TTR complex, led to a significant shift of the ligand towards the outer region of the binding site. Figure S5 shows the analog behavior of the protein backbone in the three complexes, which relaxed after removing the constraint on the C-alpha (time = 2100 ps), slightly increasing the RMSD values. This increment of RMSD doubled after relaxing the ligand, reaching a stable value of 1.5 Å, which indicates that all systems achieved an equilibrated structure. In the GC-1 complex, the new stability adapted to the ligand, which moved about 1 Å from the first heating and retained this disposition for the entire simulation. TG68 modified its pose first after the backbone relaxed, then after relaxing the ligand constraint, reached equilibrium with a similar deviation to that of the protein. In contrast, IS25 moved from the original pose of about 2.5 Å when the constraint relaxed, with a higher deviation than that of the protein. In all ligands, the deviation from the starting position, irrespective of the magnitude, led to a loss of interaction with T106. The final complexes, reported in Figure 4, revealed a strong interaction between GC-1 and the key residues (attributed to the starting polar interaction with S117 side chain during the MD simulation), whereas IS25 and TG68 adopted a conformation that lost the tight connectivity with S117, yet retained the interaction with K15. Notably, T_4_ was also reported to have tight ionic interactions with K15 and E54, but it failed to mediate the stable connectivity to S117 [2]. Taken together, the MD simulation results suggest the superior potential of GC-1 to accommodate an optimal interaction with TTR, consistent with our NMR and aggregation assay results.

**Figure 5:**
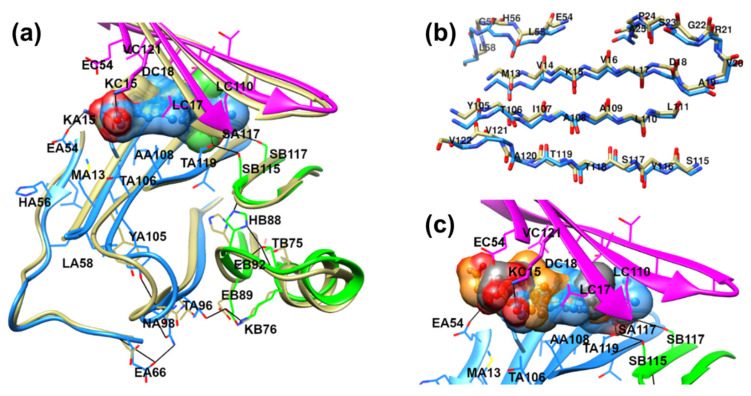
Structural accommodation of bound GC-1 on TTR residues involved in NMR perturbation, predicted through the MD simulation. The average MD poses of GC-1 (**a**) colored by heteroatoms, carbons colored light blue) are superimposed with tafamidis (**a**) colored by heteroatoms, carbons colored wheat) in the crystal structure pose (PDB 3TCT), and with IS25 and TG68 (**c**) colored by heteroatoms, carbons colored orange and gray, respectively) in their MD average pose. (**a**) Effects of GC-1 binding on the region of M13-D18, E54-L58, K76, H88-E92, and N98. The network of H-bonds is illustrated in full black lines. (**b**) Details of GC-1–TTR (light blue) and tafamidis–TTR (wheat) backbone disposition of the binding site.

A deeper analysis of the GC-1–TTR complex revealed further correlations between the MD and NMR results. GC-1 appeared to occupy the whole binding cavity, filling the region of K15 with a flexible acetic moiety, and deeply inserting the oxydril group in the central serine area (Figure 5a). The steric effect of the flexible oxyacetic moiety influenced the β-sheet in the M13-D18 and E54-L58 regions more than the planar tafamidis in the 3TCT crystallographic structure (Figure 5a,b, wheat colored). A slight deviation of the ribbon position, induced by GC-1, was evident in both chain A (Figure 5a, light blue) and C (Figure 5a, magenta). In particular, a higher variation was detected for the backbone M13-V14 and L55-L58 residues, due to the flexible oxyacetic moieties, L17-D18 and S23, induced by the methyl groups, T106-I107, A120-V121, probably due to a shift propagation through the sheets (Figure 5b). In addition, the final MD structure showed an H-bond network originating from the inner serines 115 and 117, which extended to H88, T75, E89, K76, T96, N98, E66, and Y105. In the crystal structure of the tafamidis–TTR complex (superposed to the GC-1–TTR complex in Figure 5a), the network was interrupted at different conformations of H88 (potentially due to the presence of two water molecules, usually present in crystallographic structures), and predominantly at the N98 disposition. A great deviation of the protein backbone in the tract N98-T106 was detected, which is crucial for the H-bond network in the GC-1–TTR complex, and also in a variety of TTR crystal structures [50]. All these results are in agreement with the NMR signal perturbation pattern of GC-1, compared with previous results relative to tafamidis [18]. Taken together, it was confirmed that the stronger involvement of M13-A25 and L55-L58 tracts in the GC-1–TTR complex compared to the protein bound to tafamidis, the different participation of H88 and E89 compared to the tetramer stabilization in the two complexes, and N98 plays a critical role in aligning the complexes, though this is not significant in tafamidis binding. Regarding our three thyromimetics, it was clear that the higher hindrance of IS25 and TG68 in the M13-A25 and L55-L58 regions (Figure 5c) resulted in a unique pose of the oxyacetic moiety. The superposition of GC-1 (light blue), IS25 (orange), and TG68 (gray), in Figure 5c showed a higher exposure of the GC-1 methyl towards D18, and of the TG68 acetic chain towards M13 and T106. A different NMR signal perturbation pattern among our thyromimetics was also registered for H88 and E89. An analysis of the H88, E89, and N98 occupancy during the MD simulation reported in Figure S6 highlighted the permanency of the TG68-TTR complex side chains at H88 and N98, in a conformation similar to that of TTR bound to tafamidis. In IS25-bound TTR, H88 freely rotated between the A and B monomers, and N98 switched between two positions, both directing the NH_2_ moiety towards E98. Additionally, GC-1 induced a populated conformation of H88 facing the B monomer, whereas the amidic group of N98 flipped directing the NH_2_ moiety equally towards monomer A and monomer B. In addition, the NMR signal perturbation pattern was in agreement with our MD simulation results, and the H88 behavior traced the latest biophysical studies on TTR amyloidogenesis [51].

## 3. Discussion

Thyromimetics are novel and promising therapeutic molecules with versatile activities. Therefore, numerous molecules originating from TH, TH metabolites, or TH derivatives, have been developed, and their working mechanisms extensively studied. However, other than knowing that TTR is the native transporter of TH, the potential effects of most thyromimetic molecules on TTR have not been studied in detail. It is notable that the X-ray crystallographic study was previously conducted to reveal the structural features of the GC-1−TTR complex [43], yet further studies to appreciate the molecular details of this interaction or for the other related ligands have still been lacking. In this study, we investigated whether GC-1 and the recently developed thyromimetics IS25 and TG68 interact with TTR. The NMR spectroscopic and computational data showed that these ligands bind to the hydrophobic pockets of TTR, where T_4_ or tafamidis binds [2,13]. Specifically, among the thyromimetics we tested, GC-1 induced the most significant signal perturbation in the NMR titration experiment, and formed the most favorable complex with TTR, predicted from the docking simulation. Our TTR aggregation assays provided consistent results showing that GC-1 is the most efficient inhibitor of TTR amyloidosis. 

While the superior activities of GC-1 were not clearly appreciated by the previous X-ray crystallographic study [43], our data provide better clues to explain it. First, we identified that the interaction interface between GC-1 and TTR covers most of the residues, such as K15, T106, and S117, which have been reported to participate in the interaction with T_4_, tafamidis, or iododiflunisal [2,13,49]. In particular, our observation is more consistent with the direct interaction of S117 with GC-1, distinctive with the X-ray crystallographic model where a water molecule mediates the interaction between GC-1 and S117. The NMR signal perturbation showed that the residues located deep inside the binding pocket, such as S115, S117, T118, and A120, were significantly affected by GC-1, supporting the docking simulation results of GC-1 displacing a water molecule and forming a direct hydrogen bond with S117. In addition, the docking simulation predicted both the distance between the hydroxyl group of GC-1 and the hydroxyl group of S117, and the distance between the carboxylate of GC-1 and the terminal amine of K15 are in the closest proximity when compared with the binding modes for T_4_, tafamidis, IS25, and TG68. This was further corroborated by both the NMR signal perturbation results, where less noticeable shifts in signal occurred upon addition of IS25 or TG68, and the TTR aggregation assays, where less significant inhibitory effects were noticed for IS25 and TG68, compared to GC-1. Moreover, GC-1 may induce additional structural changes in TTR through enhanced the interaction with the G53-T59 residues. The X-ray structural model of the GC-1−TTR complex did not exhibit any structural changes in this region [43], and previous NMR spectroscopic studies regarding the binding of tafamidis to TTR also did not report signal shifts in the same region [18,47]. In contrast, our NMR and docking simulation data indicate that the interaction between the terminal carboxylate of GC-1 and K15 may cause subsequent structural perturbation of the ionic interactions between K15, E54, and H56. This raises the intriguing possibility that GC-1 engages in a wide interaction interface with TTR due to the fact that it only requires minor yet effective structural rearrangements. Notably, several chemical chaperone molecules, such as epigallocatechin gallate (EGCG) and the related natural flavonoids, were reported to bind to TTR without occupying the T_4_ binding sites, and stabilize the TTR tetramer [52,53,54,55]. The efficacy of these molecules was tested in a mouse model [56] and in the observational studies of human patients [57,58], indicating that these molecules may constitute a novel repertoire for TTR amyloidosis therapeutics. Intriguingly, the X-ray crystallographic model of the TTR-EGCG complex showed that one of the binding sites for EGCG is close to the residues T49-G53 [53], where we proposed to undergo structural changes by GC-1 binding. This coincidence raises a notable question whether the binding of EGCG and GC-1 will incur synergistic structural rearrangement in the region around the T49-T59 residues. Subsequent structural and biochemical studies need to be conducted to investigate possible structural changes induced by thyromimetic ligands and chemical chaperones. 

A few ambiguities of this study remain to be elucidated. First, it appears clear that all the tested compounds form a stable complex with TTR, yet detailed structural characterizations of each complex are necessary to appreciate the molecular interactions. In particular, we observed a significant number of NMR signals that broadened out in the ligand titration, suggesting that the ligand complex comprises considerable dynamic processes. The dynamic ligand interaction modes in the T_4_ binding pocket of TTR have been observed previously [2,59], and our calculations indicate the possibility of multiple binding modes for the thyromimetic molecules tested here. Indeed, the structural heterogeneity of GC-1 in the binding site of TTR as observed in the previous X-ray crystallographic study and the present work may give an additional clue for multiple binding modes of thyromimetic ligands. Because NMR spectroscopic observation is based on a solution-state sample, not being restricted due to crystal packing or non-native crystallization condition, our approaches may constitute an additional and advantageous view to appreciate the structural diversity of TTR and its ligand molecules [60,61]. Therefore, subsequent studies are necessary to appreciate the detailed dynamics in the ligand-bound TTR and its potential effects as a potent aggregation inhibitor. In addition, it should be stressed that this study needs to be followed by additional in vitro and in vivo experiments considering various aspects of TTR amyloidosis and the related pathology. For example, it was proposed that amyloidogenic variants of TTR may have different structural states (e.g., deformed T_4_ binding sites), and accordingly, distinctive pathogenic mechanisms [62]. This suggests that the responses to therapeutic molecules may differentiate between TTR variants, for which we are also currently conducting a few follow-up studies. In addition, the proteolysis-induced fragmentation of TTR was recently proposed as an efficient mechanism facilitating TTR amyloidosis even in a physiological pH [19]. Although the kinetic stabilizers, such as tafamidis and mds84, were shown to inhibit the proteolytic fragmentation of TTR, the detailed mechanism behind this is still elusive [20]. Therefore, the in vivo and in vitro protective effects of GC-1, IS25, and TG68 against TTR proteolysis need to be evaluated along with detailed structural characterization. Moreover, it will be essential to validate the multi-functionality and overall efficacy of these thyromimetics in suitable model systems. For example, we propose that their high affinity toward TTR may compromise their functionality as effective TRβ activators, resulting in reduced multi-functional activity as a therapeutic molecule for TTR amyloidosis, or related pathologies. Therefore, thyromimetics with different scaffolds need to be considered to incur synergistic effects with existing therapeutics.

Taken together, the present study showed that diphenyl-methane-based thyromimetics are capable of inhibiting TTR aggregation, potentially preventing the subsequent pathogenic processes. Simulations showed that GC-1 has the greatest potential among the thyromimetics studied to interact with TTR as it requires the least structural modifications to accommodate GC-1–TTR’s complex formation. Based on their TH-like activities and efficacy as TTR stabilizers in Aβ-related pathologies, we propose that GC-1, together with its analogs, IS25 and TG68, could be novel candidates as multi-functional drugs for TTR amyloidosis, and related pathologies. 

## 4. Materials and Methods

### 4.1. Sample Preparation

Recombinant human TTR samples were prepared as described previously [63]. Briefly, the pQE30 vector (Qiagen, Hilden, Germany) for human TTR expression was transformed into M15(pREP4) *Escherichia coli* competent cells, which were subsequently grown in LB media for unlabeled protein samples, or in M9 minimal media for isotopically labeled samples. As the tetrameric complex of TTR is relatively large (~55 kDa), we employed the fractional deuteration protocol, along with the ^13^C and ^15^N labeling procedures, to complement signal broadening in the NMR spectra. For this, we used the M9 minimal media that was prepared with 3 g/L [U-^13^C]-D-glucose and 0.5 g/L ^15^NH_4_Cl in D_2_O (Cambridge Isotope Laboratories, Tewksbury, MA, USA). We confirmed with NMR that most Hα, a significant portion of Hβ, and some portions of the other protons were efficiently deuterated, resulting in a notable decrease in the ^1^H-^15^N signal width for TTR.

The subsequent sample purification was initiated by sonication of the cell pellet. After cell lysis, the cell debris was removed by centrifugation at 20,000 rpm (4 °C for 40 min), and the supernatant was applied to an anion exchange column (HiTrap Q HP; Cytiva, Marlborough, MA, USA) that was implemented in the ÄKTA FPLC system (Cytiva). The TTR-containing fractions were obtained in the NaCl gradient elution step, which was further checked by SDS-PAGE analysis. The pulled fraction was subsequently concentrated and applied to a gel filtration column (HiLoad 16/600 Superdex 75 pg; Cytiva). The fractions were analyzed by SDS-PAGE for purity check. The pure fractions were finally combined, concentrated, and flash-frozen with liquid N_2_, and stored in a –80 °C freezer.

### 4.2. TTR Aggregation Assay

For the acid-induced aggregation assay, we followed the protocol described previously [4,64]. Briefly, TTR samples prepared in PBS buffer pH 7.4 (10 mM phosphate, 140 mM NaCl, 2.7 mM KCl) were first mixed with either DMSO, or equimolar, and five-fold test compounds (GC-1, IS25, or TG68) dissolved in DMSO. These TTR-compound mixtures were then mixed 1:1 with acetate buffer (200 mM sodium acetate pH 4.2, 100 mM KCl, 1 mM EDTA) to make the final solution of pH 4.4 [64]. The final monomer concentration of TTR was 64 μM. The mixtures were incubated at 37 °C for four days without agitation. 

The aggregation level was quantified either by ThT fluorescence or turbidity, both of which were measured with a Tecan Spark™ 10M microplate reader (Männedorf, Switzerland). For ThT fluorescence measurement, the aggregation mixtures were first diluted to the final concentration of 4 μM TTR with a buffer (200 mM Tris pH 8.0, and 150 mM NaCl). Next, 2 μL 2 mM ThT stock solution in 200 mM Tris pH 8.0, 150 mM NaCl was added to 400 μL of the diluted solution. The final samples were vortexed and transferred to a 96-well black-wall microplate in triplicate for the ThT fluorescence measurement (excitation and emission wavelengths were set to 440 nm and 482 nm, respectively). The average fluorescence values were reported as mean ± SD from triplicate measurements. For the turbidity measurement, the aggregation mixtures were vortexed and pipetted into a 96-well UV-compatible transparent microplate in triplicate. The turbidity of the samples was measured by recording the optical density at 330 nm, and the mean ± SD was reported. We confirmed the consistency of the ThT fluorescence and turbidity results by repeating the measurements several times.

### 4.3. NMR Spectroscopy 

All the NMR experiments were conducted using an Avance III HD 850 MHz NMR spectrometer equipped with a cryogenic HCN (triple-resonance) probe (Bruker, Billerica, MA, USA). Samples of 0.25 mM TTR for NMR were prepared from partially deuterated [U-^13^C, U-^15^N] WT-TTR in a buffer of 50 mM MES pH 6.5, 100 mM NaCl, 5 mM dithiothreitol, 0.01% NaN_3_, and 7% D_2_O. Thereafter, a 300 μL sample was added to 5-mm Shigemi tubes (Sigma-Aldrich, St. Louis, MO, USA). The signal assignment information of WT-TTR was first obtained from BMRB accession numbers 5507 [65] and 27514 [62], and a few ambiguities were subsequently resolved by obtaining and analyzing 2D ^1^H-^15^N TROSY-HSQC, 3D TROSY-HNCA, and 3D ^15^N-NOESY-HSQC spectra. NMRFAM-Sparky software was used for signal assignment and NMR data analysis [66].

For the subsequent titration experiments, the thyromimetic compounds dissolved in DMSO-d_6_ were added serially to the TTR samples, and 2D ^1^H-^15^N TROSY-HSQC spectra of each mixture were obtained. Up to three-fold compounds were added, for which 2D ^1^H-^15^N TROSY-HSQC and 3D TROSY-HNCA spectra were acquired. The 3D TROSY-HNCA spectrum was useful for resolving the overlapped signals in the 2D spectrum. We observed that further addition of thyromimetic compounds did not cause any additional spectral changes in TTR. We also confirmed that the same amount of DMSO-d_6_ without any compound did not cause a signal perturbation in the NMR spectrum of TTR.

The backbone ^1^H-^15^N signal perturbation (Δδ_NH_) of WT-TTR by the thyromimetic ligands was calculated for each residue using the following equation: Δδ_NH_ = [(Δδ_N_)^2^/5 + (Δδ_H_)^2^]^1/2^, where Δδ_N_ and Δδ_H_ are the chemical shift differences of the ^1^H-^15^N signals between the free and ligand-bound states of WT TTR. In the signal perturbation plot, the residues whose signals broadened due to ligand addition are marked with red triangles, whereas the proline residues that do not show any ^1^H-^15^N signal or the residues whose signals could not be followed due to signal overlap were left blank in the plot.

### 4.4. Docking Simulation

The TTR-T_4_ complex 2ROX [2] and TTR-GC1 complex 3NEE [38] were selected from the Protein Data Bank [67] and transformed into a tetramer using the Higher-Order Structure tool of the Chimera program (San Francisco, CA, USA) [68]. Missing hydrogen atoms were added to the proteins using the Maestro program (Schrödinger, New York, NY, USA) [69], according to the predicted protonation state at pH 7.0, and His residues were set to their δ-protonation state. Asn, Gln, and His residues were absent in the range of interest for docking; therefore, no flip corrections were necessary. The region of interest for docking, performed using the GOLD program (Cambridge Crystallographic Data Centre, Cambridge, UK) [70], was defined in such a manner that both proteins contained all the residues within 10 Å of the original ligands. The “allow early termination” command was deactivated. All ligands were submitted to 40 Genetic Algorithm runs using ChemScore fitness functions with flexibility improvement [71], clustering the output orientations on the basis of an RMSD distance of 1.5 Å. The extra parameters of gold were used to permit S117 free side chain rotations. The default gold parameters were used for all variables.

### 4.5. Molecular Dynamics 

Complexes derived from the best docking pose of each ligand in the TTR binding site were subjected to molecular dynamics. The simulation, aimed to test the stability of the predicted pose, was performed using AMBER Version 14 (San Francisco, CA, USA) [72]. The complexes were placed in a rectangular parallelepiped water-box, using an explicit solvent model for water (TIP3P); the complexes were solvated with a 10 Å water cap. Sodium ions were added as counterions to neutralize the system. Prior to MD simulations, three steps of minimization were carried out: first, optimizing the solvent, then relaxing the complex, and then the ligand. Particle mesh Ewald electrostatics and periodic boundary conditions were used in the simulation. The MD trajectories were run using the minimized structures as starting conformations. The time step of the simulations was 2.0 fs with a cutoff of 10 Å for the non-bonded interaction, and SHAKE was employed to keep all bonds involving hydrogen atoms rigid. The constant-volume periodic boundary MD was carried out for 500 ps, during which the temperature was raised from 0 to 300 K. Then, 9.5 ns of constant-pressure periodic boundary MD was carried out at 300 K, using the Langevin thermostat to maintain the temperature of our system constant, constraining all the α carbons, and ligand, with 10 kcal of constant harmonic force in the first 1600 ps; successively, the ligand was relaxed after further 1600 ps, and the last 6.3 ns of simulation were performed without any constraint. General Amber force field parameters were assigned to the ligands, while partial charges were calculated using the AM1-BCC method. The MD trajectories were analyzed using the MD Movie tool of Chimera [68], and the cpptraj module of AMBER 14 [73].

## Supplementary Materials

Supplementary materials can be found at https://www.mdpi.com/article/10.3390/ijms22073488/s1.

## Abbreviations

[U-^13^N, U-^15^N]	Uniformly ^13^C- and ^15^N-labeled
BBB	Blood brain barrier
BMRB	Biological Magnetic Resonance Data Bank
DMSO	Dimethyl sulfoxide
EDTA	Ethylenediaminetetraacetic acid
GC-1 HSQC MD	Sobiterome Heteronuclear single quantum coherence Molecular dynamics
MES	2-(N-morpholino)-ethanesulfonic acid
NMR	Nuclear magnetic resonance
PDB	Protein data bank
RMSD TH	Root-mean-square deviation Thyroid hormone
ThT	Thioflavin T
TROSY	Transverse relaxation-optimized spectroscopy
TRβ	Thyroid hormone receptor β
TTR	Transthyretin
WT	Wild-type
