# Supplementary material for: Diphenyl-Methane Based Thyromimetic Inhibitors for Transthyretin Amyloidosis
Source: Int J Mol Sci. 2021 Mar 28;22(7):3488. doi: 10.3390/ijms22073488 (PMC8038088; doi:10.3390/ijms22073488)
Supplement: Supplementary file 1 [file ijms-22-03488-s001.pdf]

Supplementary Figures

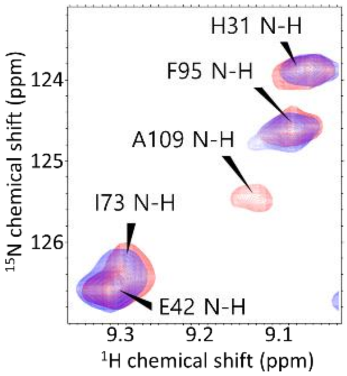

Figure S1. Presentative region of the ^{1}H-^{15}N HSQC spectrum for isotopically labeled-TTR in the presence (blue) or absence (red) of GC-1. The residues of TTR corresponding to each signal are denoted with a black bar. Note that GC-1 incurred signal broadening for A109, while the other residues experienced slight or no signal shift.

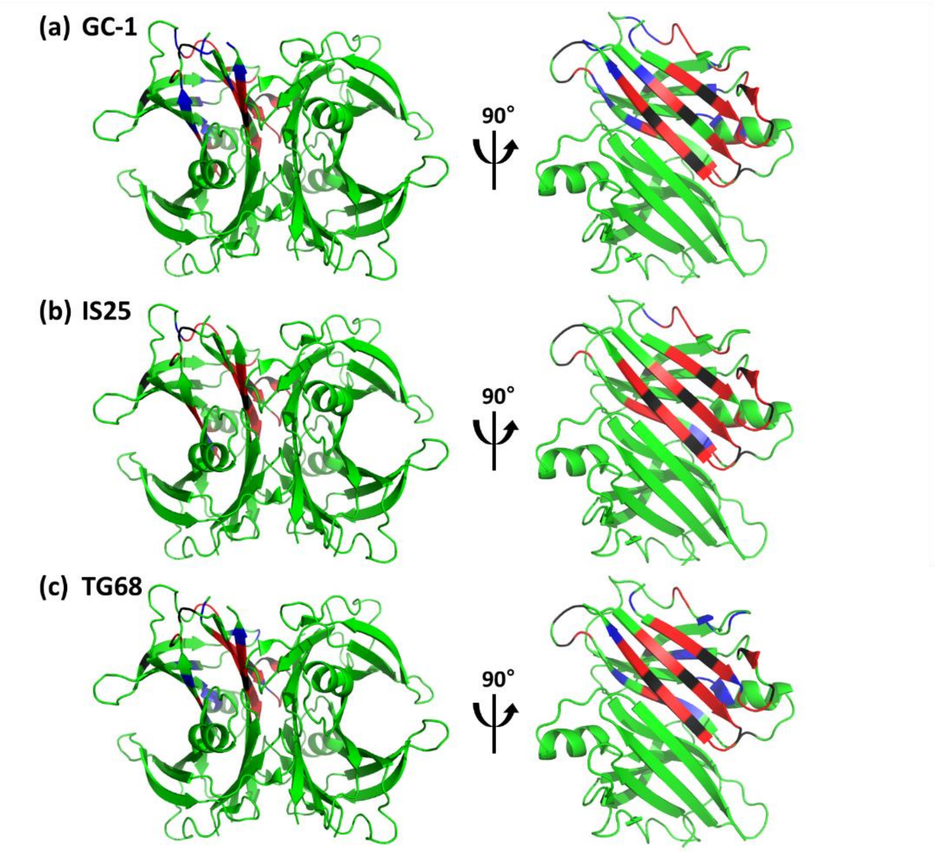

Figure S2. ^{1}H-^{15}N HSQC signal perturbation results of TTR in the titration of GC-1 (**a**), IS25 (**b**), and TG68 (**c**) were mapped onto the structural model of TTR (PDB 4TLT). The residues whose signals broadened out are colored red, while the residues whose signals exhibited significant shifts during the titration are colored blue (see Figure 3). Prolines, which do not show an ^{1}H-^{15}N signal, and the residues, whose signals cannot be assigned (due to spectral ambiguity such as signal overlapping), are colored black. The color mapping was first made on one subunit of the homotetrameric complex (left), while the dimeric structure exposing the hydrophobic binding pockets was used in the 90°-rotated figures (right) for better visibility of the NMR signal perturbation results.

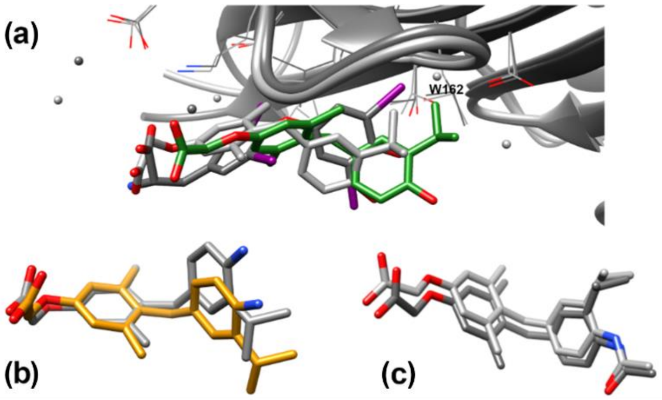

Figure S3. Comparison of the best docking poses of GC-1, IS25, and TG68, which were calculated from the 3NEE and 2ROX structures. (**a**) Superposition of 3NEE (light grey), 2ROX (dark grey) binding sites and the best pose of GC-1 (green) obtained by a docking simulation with the 2ROX structure. (**b**) Superposition (RMSD 2 Å) of the best pose of IS25 obtained by a docking simulation with the 2ROX (orange) and 3NEE (light grey) structures; (**c**) Superposition (RMSD 0.9 Å) of the best pose of TG68 obtained by a docking simulation with the 2ROX (dark grey) and 3NEE (light grey) structures.

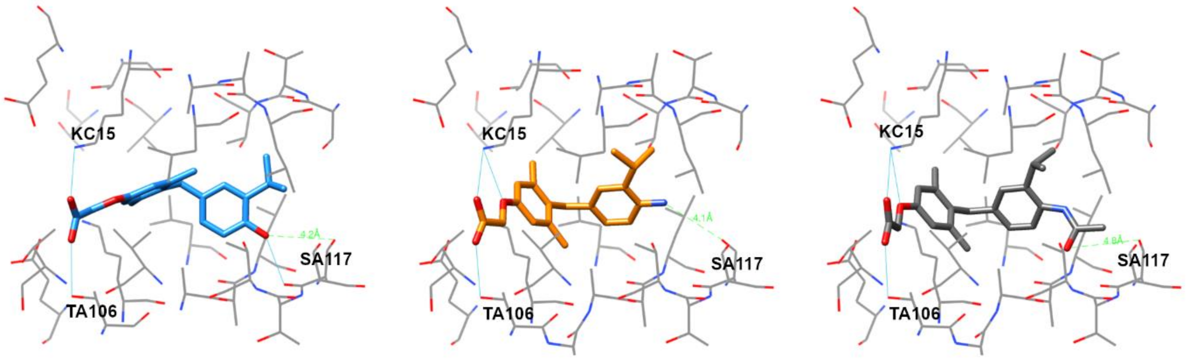

Figure S4. Results of the docking simulation of GC-1 (left), IS25 (middle), and TG68 (right), in TTR. Residues directly involved in polar interactions are labelled. The binding site is located at the dimer–dimer interface (specular monomers A and C are reported), and have a funnel-shaped pseudo symmetric morphology, with polar residues placed at the entrance, a hydrophobic core located at the center, and hydrophilic residues on the inside.

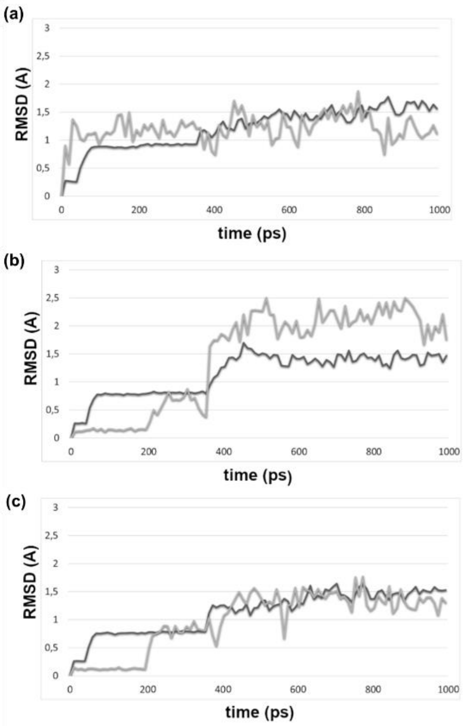

Figure S5. RMSD of the alpha-carbons of the protein (dark colored line) and the heavy atoms of the ligand (gray colored line) during the MD simulation of (**a**) GC-1, (**b**) IS25, (**c**) TG68 complexes, respectively.

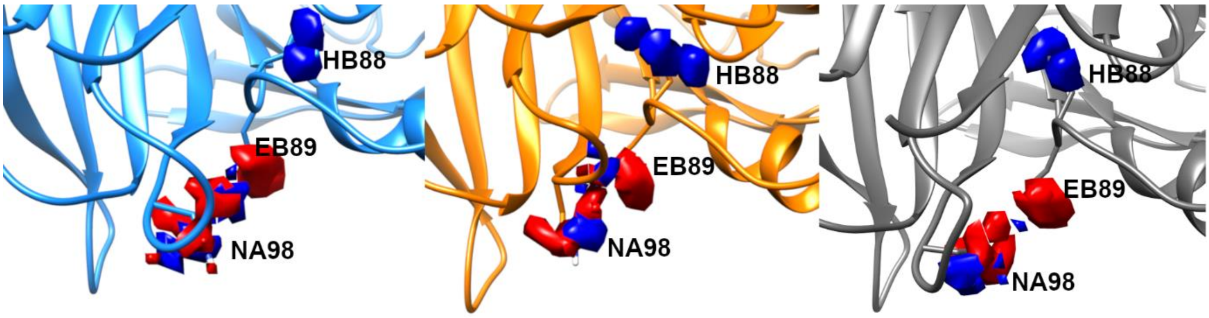

Figure S6. Regions of occupancy at the heteroatom side chains of H88, E89 and N98, during the MD simulations of TTR bound to GC-1 (left), IS25 (middle), and TG68 (right). Protein coordinates are casually selected within free MD frames. Occupancy of oxygens and nitrogens are colored red and blue, respectively.
